# Luteolin Targets the Toll-Like Receptor Signaling Pathway in Prevention of Hepatic and Adipocyte Fibrosis and Insulin Resistance in Diet-Induced Obese Mice

**DOI:** 10.3390/nu10101415

**Published:** 2018-10-03

**Authors:** Eun-Young Kwon, Myung-Sook Choi

**Affiliations:** 1 Department of Food Science and Nutrition, Kyungpook National University, 1370 San-Kyuk Dong Puk-Ku, Daegu 41566, Korea; savage20@naver.com; 2 Center for Food and Nutritional Genomics Research, Kyungpook National University, 1370 San-Kyuk Dong Puk-Ku, Daegu 41566, Korea

**Keywords:** fibrosis, inflammation, luteolin, obesity, Toll-like signaling pathway

## Abstract

This study was to investigate the protective role of luteolin on inflammation-mediated metabolic diseases, focusing on the role of luteolin in the modulation of the Toll-like receptor (TLR) signaling pathway. C57BL/6J mice were fed a normal, high-fat, or high-fat + 0.005% (*w*/*w*) luteolin diet for 16 weeks. Luteolin improved chronic low-grade inflammation by modulating the TLR signaling pathway, resulting in reduced pro-inflammatory cytokines and macrophage accumulation. A positive relationship was detected between gene expressions of *Tlr5*, *Map2k7*, *Mapk12*, *Mapk13*, and *Mapk9* and lipogenesis in epididymal white adipose tissue (eWAT) of luteolin-treated mice, which was linked to attenuation of hepatic lipotoxicity by increasing free fatty acid (FFA) flux to the WAT. Luteolin prevented fibrosis by decreasing extracellular matrix accumulation and cathepsin gene expressions, while enhancing the hepatic antioxidant system. *Emr1* and *Ccl7*, important markers inducing low-grade inflammation, were affected by advanced age and greater body weight, which were normalized by luteolin treatment. Luteolin improved insulin resistance by normalizing pancreatic islet dysfunction and differentially modulating the plasma glucagon-like peptide-1 and gastric inhibitory polypeptide levels. Our results suggest that luteolin ameliorates diet-induced obesity and its comorbidities. Overall, this study provides novel insights into the effect of luteolin on the links among adiposopathy, insulin resistance, hepatic steatosis, and fibrosis.

## 1. Introduction

Obesity is defined as excessive fat accumulation and can lead to metabolic complications, including dyslipidemia, adipokines dysregulation, insulin resistance (IR), inflammation, fibrosis, and steatohepatitis. White adipose tissue (WAT) is responsible for the storage of energy in the form of triacylglycerol, and thus protects other organs and tissues from ectopic fat accumulation resulting in lipotoxicity. WAT is also a critical endocrine organ capable of controlling the energy homeostasis and metabolism of several tissues through the secretion of several adipokines/cytokines such as leptin, adiponectin, and TNF-α that are known to mediate lipid metabolism, inflammation, and IR [1].

Obesity promotes not only adipocyte hypertrophy but also WAT macrophage accumulation, which can contribute to inflammation; this then triggers the production and deposition of extracellular matrix (ECM), including collagen, to induce adipose tissue fibrosis, possibly as a result of the pro-inflammatory adipokines/cytokines released by adipocytes and infiltrated macrophages [2]. In obesity, adipose tissue inflammation is further exacerbated by the excessive accumulation of ECM components resulting in the pathogenesis of fibrosis. Moreover, accumulating evidence indicates that some cathepsins act as a link between adipose tissue and fibrosis in obesity through proteolysis of ECM components [3,4,5,6,7]. However, the underlying mechanism by which the interactions of adipocyte hypertrophy, adipokine dysregulation, ECM accumulation, and inflammation lead to the development of adipose tissue fibrosis, IR, and hepatic steatosis has yet to be fully established, especially at the level of the transcriptional changes that occur in obese adipose tissue.

Luteolin (LU, 3′,4′,5,7-tetrahydroxyflavone) is a flavone found in leaves, vegetables, fruits, and natural herbal drugs such as parsley, thyme, peppermint, and celery. Some of the proven biological effects of LU are a variety of anti-inflammatory actions. However, little is known about the detailed mechanisms linked to the anti-metabolic syndrome action of luteolin based on the integration of transcriptional profile and phenotypic biomarkers. Accordingly, the aim of this study was to investigate the protective role of oral luteolin treatment on inflammation-mediated metabolic diseases in diet-induced obese C57BL/6J mice, focusing on its role in modulating the Toll-like receptor (TLR)-signaling-pathway-triggered up-regulation of pro-inflammatory cytokines. C57BL/6J is the most widely used inbred strain and is susceptible to diet-induced obesity, type 2 diabetes, and atherosclerosis. These results should provide novel insights into the effect of luteolin on the links among adiposopathy, IR, hepatic steatosis, and fibrosis.

## 2. Materials and Methods

### 2.1. Experimental Animals and Diets 17.65 82.35

Four-week-old male C57BL/6J mice were purchased from the Jackson Laboratory (Bar Harbor, ME, USA). The 36 mice were individually housed at room temperature (24 °C) and under a 12 h light/dark cycle, and were fed the AIN-76 semipurified diet for a one-week acclimation period after arrival. After acclimation, the mice were randomly divided into three groups consisting of normal diet (ND, *n* = 10), high-fat diet (HFD, *n* = 13), and HFD with 0.005% (*w*/*w*) luteolin (Sigma Chemicals, St. Louis, MO, USA) (LU, *n* = 13) for 16 weeks. The HFD is the AIN-76 semipurified diet containing 40 kcal% fat by calories: 15% (*w*/*w*) corn oil and 85% (*w*/*w*) lard of total fat. All experimental diets were prepared every week and stored in a dark room at −4 °C. Body weight and blood glucose levels were measured every 1 and 2 weeks, respectively. At the end of the experimental period, all mice were anesthetized with ether after a 12 h fast. Blood was taken from the inferior vena cava for the determination of glucose, plasma lipid, and hormone concentrations. The liver and adipose tissues were removed, rinsed with physiological saline, weighed, immediately frozen in liquid nitrogen, and stored at −70 °C until analysis. The study was performed under protocols approved by the Ethics Committee at Kyungpook National University (approval No. KNU 2010-4-14).

### 2.2. Blood Glucose, IPGTT, and Plasma Biomarkers

Every 2 weeks, the 12 h fasting blood glucose level was measured from tail vein blood with a glucose analyzer (GlucoDr SuperSensor, Allmedicus, Anyang, Korea). For the IPGTT analysis performed in the 15th week after the start of the diet experiments, mice fasted for 12 h were injected intraperitoneally with glucose (0.5 g/kg body weight), and the blood glucose level was determined in the tail vein blood at 0, 30, 60, and 120 min later. Plasma biomarkers were measured using fluorescent magnetic bead-based assays (Bio-Rad, Millipore, Hercules, CA, USA): insulin, glucagon, leptin, resistin, plasminogen activator inhibitor-1 (PAI-1), glucagon-like peptide-1 (GLP-1) and gastric inhibitory polypeptide (GIP) (Bio-Plex Pro™ mouse diabetes assays), IL-6, TNF-α, monocyte chemoattractant protein-1 (MCP-1) and macrophage inflammatory protein 1 beta (MIP-1β) (Bio-Plex Pro™ mouse cytokine assays), and adiponectin (Bio-Plex Pro™ mouse diabetes adiponectin assay). The plasma adipsin (R&D Systems, Minneapolis, MN, USA), soluble CD antigen 163 (sCD163; Mybiosource, San Diego, CA, USA), glutamic oxaloacetic transaminase (GOT) (Asan, Seoul, Korea), and glutamic pyruvic transaminase (GPT) (Asan, Seoul, Korea) levels were analyzed using a commercially available kit.

### 2.3. Plasma and Hepatic Free Fatty Acid (FFA) Measurements

The plasma and hepatic FFA levels were both measured using an enzymatic assay kit (Wako Chemicals, Richmond, VA, USA), except that the hepatic FFA level was determined after extraction according to the methods of Folch [8].

### 2.4. Histopathological Analysis

The liver, epididymal adipose, and pancreas tissues were eliminated from each mouse. The liver and epididymal adipose tissue samples were subsequently fixed in 10% (*v*/*v*) paraformaldehyde/PBS and embedded in paraffin for immunohistochemistry staining for CD68 and MCP-1 and Masson’s trichrome staining. The pancreas tissue sample was fixed in 4% (*v*/*v*) paraformaldehyde/PBS and embedded in paraffin for immunohistochemistry staining of insulin and glucagon. Stained slices were viewed under a microscope set at 200× magnification.

### 2.5. Hepatic Enzyme Activities and Lipotoxicity Markers

The enzyme source fraction in the liver was prepared as previously described [9] and the protein concentrations were determined using the Bradford method. Catalase (CAT) [10], superoxide dismutase (SOD) [11], glutathione peroxidase (GPx) [12], and glutathione reductase (GR) [13] activities were measured as previously described. The hepatic H_2_O_2_ [14] and thiobarbituric acid reactive substances (TBARS) [15] levels were also determined as previously described.

### 2.6. RNA Preparation and Quality Control

Total RNA was extracted from adipose tissue using TRIZOL reagent (Invitrogen Life Technologies, Grand Island, NY, USA), and three pooled RNA sample sets were then constructed for the ND, HFD, and LU groups as previously described [16]. RNA was stored at −70 °C prior to further analysis by microarray and RT-qPCR.

### 2.7. RT-qPCR

Total RNA (1 μg) was used to synthesize cDNA via the QuantiTect Reverse Transcription kit (QIAGEN Gmbh, Hilden, Germany), and mRNA expression was quantified by RT-qPCR using the SYBR green PCR kit (Qiagen, Hilden, Germany) and the CFX96TM real-time system (BIO-RAD, Hercules, CA, USA). Gene-specific mouse primers were used as indicated in Table S1. Ct values were normalized to the reference gene GAPDH and the relative gene expression was calculated with the 2^−ΔΔ*C*t^ method [17].

### 2.8. Microarray Analysis

Total RNA was amplified and purified using the Ambion Illumina RNA amplification kit (Ambion, Austin, TX, USA). Biotinylated cRNA (750 ng per sample) was hybridized to Illumina MouseWG-6 v2 Expression BeadChips (Illumina, San Diego, CA, USA) according to the manufacturer’s instructions. Hybridized arrays were washed and stained with Amersham Fluorolink streptavidin-Cy3 (GE Healthcare Bio-Sciences, Little Chalfont, UK) following the standard protocol provided in the bead array manual. Hybridization quality and overall chip performance were determined by visual inspection of both the internal quality controls and the raw scanned data in the Illumina BeadStudio software. Probe signal intensities were quantile-normalized and log-transformed. Microarray analysis was performed in the ArrayAssists software (Stratagene, La Jolla, CA, USA) and with the R programing language.

The statistical differential gene expression analysis between groups was performed using the nonparametric Rank Prod approach. Oligonucleotides showing changes between groups with a false discovery rate lower than 0.05 were considered significant. The Kyoto Encyclopedia of Genes and Genomes (KEGG) pathways mapper (www.genome.jp/kegg) was consulted for the analysis of gene functions involved in inflammation. This microarray data was deposited in the Gene Expression Omnibus (GEO) database (GEO accession numbers: GSE111412).

### 2.9. Statistical Analysis

All data are presented as mean ± standard error of the mean (SEM) and were analyzed using SPSS (SPSS Inc. Chicago, IL, USA). Statistical differences between groups (HFD and ND, and LU and HFD) were determined using Student’s *t*-test. Differences were considered statistically significant at *p* < 0.05.

## 3. Results

### 3.1. Luteolin Lowered Body Fat in Diet-Induced Obese (DIO) Mice

Luteolin treatment significantly reduced the body weight gain and weights of WAT, including depots of epididymal, perirenal, retroperitoneal, mesenteric, subcutaneous, and interscapular WAT, compared with the HFD group. Luteolin also slightly decreased the liver weight, but there was no significant difference between the HFD and LU groups (Table 1). Plasma and hepatic FFA levels were meaningfully lowered by luteolin treatment compared with those of the HFD group.

### 3.2. Luteolin Reduced Macrophage Infiltration and Fibrosis by Modulating the Inflammatory Response and Activities of Hepatic Antioxidant Enzymes in DIO Mice

**Figure 1:**
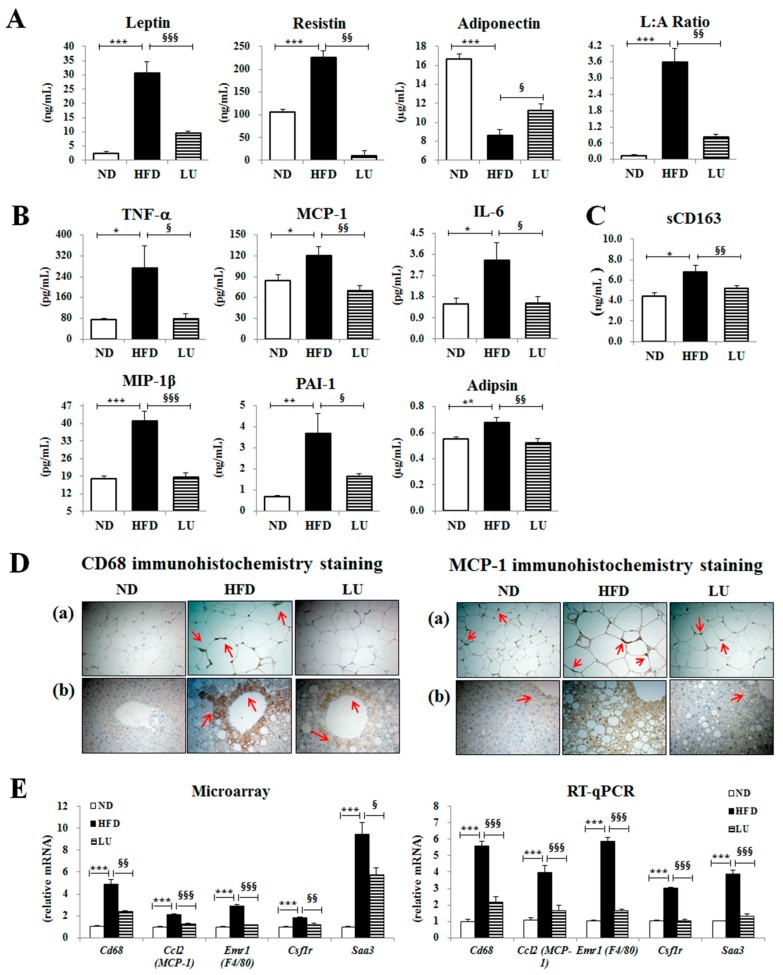
Effect of luteolin treatment for 16 weeks on the plasma adipokine/cytokine levels and macrophage-related markers in C57BL/6J mice fed the high-fat diet. (**A**) Levels of plasma adipokine leptin, resistin, and adiponectin, and the leptin/adiponectin ratio (L:A ratio). (**B**) Levels of plasma cytokines, TNF-α, MCP-1, IL-6, MIP-1β, PAI-1, and adipsin. (**C**) Plasma level of the macrophage low-grade inflammation marker sCD163. (**D**) Immunohistochemistry staining for CD68 and MCP-1 of epididymal and liver sections (200× magnification). (**E**) Adipocyte gene expression levels of *Cd68*, *Ccl2*, *Emr1*, *Csf1r*, and *Saa3*. Data are shown as means ± SEM. ND vs HFD: * *p* < 0.05, ** *p* < 0.01, *** *p* < 0.001. HFD vs LU: ^§^ *p* < 0.05, ^§§^ *p* < 0.01, ^§§§^ *p* < 0.001, ND, normal diet, HFD, high-fat diet, LU, Luteolin.

Luteolin treatment markedly decreased the plasma leptin and resistin levels and the leptin/adiponectin ratio, but significantly enhanced the plasma adiponectin level (Figure 1A). Furthermore, the levels of pro-inflammatory cytokines, including plasma TNF-α, MCP-1, IL-6, MIP-1β, PAI-1, and adipsin, and the macrophage low-grade inflammation marker sCD163 were significantly decreased by luteolin treatment compared with those in the HFD group (Figure 1B,C). The immunohistochemical staining revealed that CD68 and MCP-1 levels were lower in both the liver and adipose tissue of the LU group than in those of the HFD group (Figure 1D). The expression of macrophage infiltration-related genes (*Cd68*, *Ccl2*, *Emr1*, *Csf14*, and *Saa3*) in adipocytes was down-regulated by luteolin treatment (Figure 1E). Microarray validation with RT-qPCR showed a consistent pattern of changes in gene expression.

**Figure 2:**
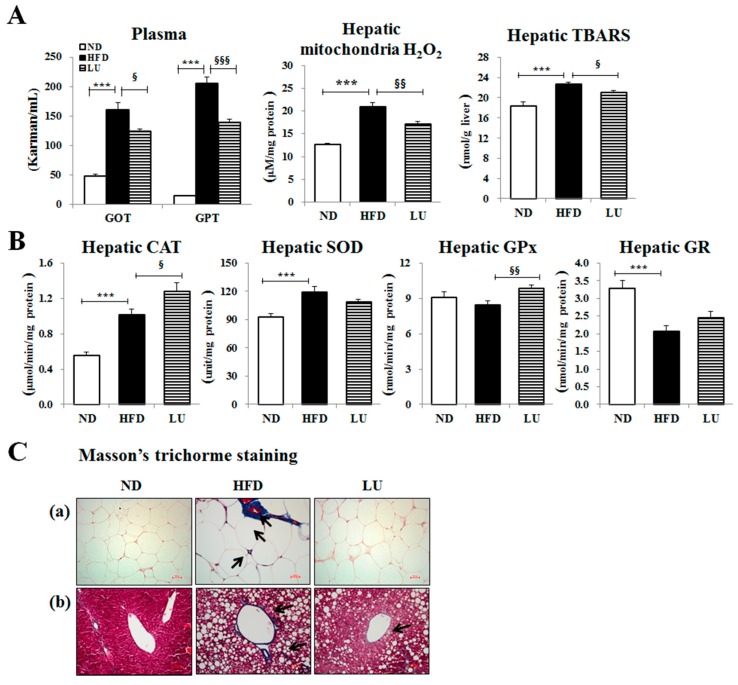
Effect of luteolin treatment for 16 weeks on hepatic lipotoxicity markers and antioxidant enzyme activities, and Masson’s trichrome staining of hepatic and adipose tissues in C57BL/6J mice fed the high-fat diet. (**A**) Plasma levels of the hepatic lipotoxicity markers GOT and GPT and the levels of hepatic mitochondrial H_2_O_2_ and TBARS. (**B**) Activities of the hepatic antioxidant enzymes CAT, SOD, GPx, and GR. (**C**) Masson’s trichrome staining of transverse liver and epididymal sections (200× magnification). Data are shown as means ± SEM. ND vs HFD: * *p* < 0.05, ** *p* <0.01, *** *p* < 0.001. HFD vs LU: ^§^ *p* < 0.05, ^§§^ *p* < 0.01, ^§§§^ *p* < 0.001.

The levels of the hepatic lipotoxicity markers, including plasma GOT and GPT, and hepatic mitochondrial H_2_O_2_ and TBARS were markedly lower in the LU group than in the HFD group (Figure 2A). The activities of the hepatic antioxidant enzymes CAT and GPx were significantly higher in the LU group compared with those in the HFD group (Figure 2B). To assess tissue fibrosis, Masson’s trichrome staining was performed, which stains the fibrous tissues containing collagen blue in order to display the fibrous tissue of the liver and adipose tissue of HFD-fed mice. The liver of the HFD group was cirrhotic and the portal vein was surrounded by fibrous bands. In addition, the epididymal WAT depot of the HFD group exhibited very pronounced trichrome-positive “streaks” interspersed among the adipocytes. By contrast, the liver of the LU group had a normal architecture and fat pads, with densely packed and very thin collagen sheets surrounding the portal vein and each adipocyte (Figure 2C).

### 3.3. Luteolin Reduced IR and Normalized the Pancreatic Beta-Cell Mass in DIO Mice

**Figure 3:**
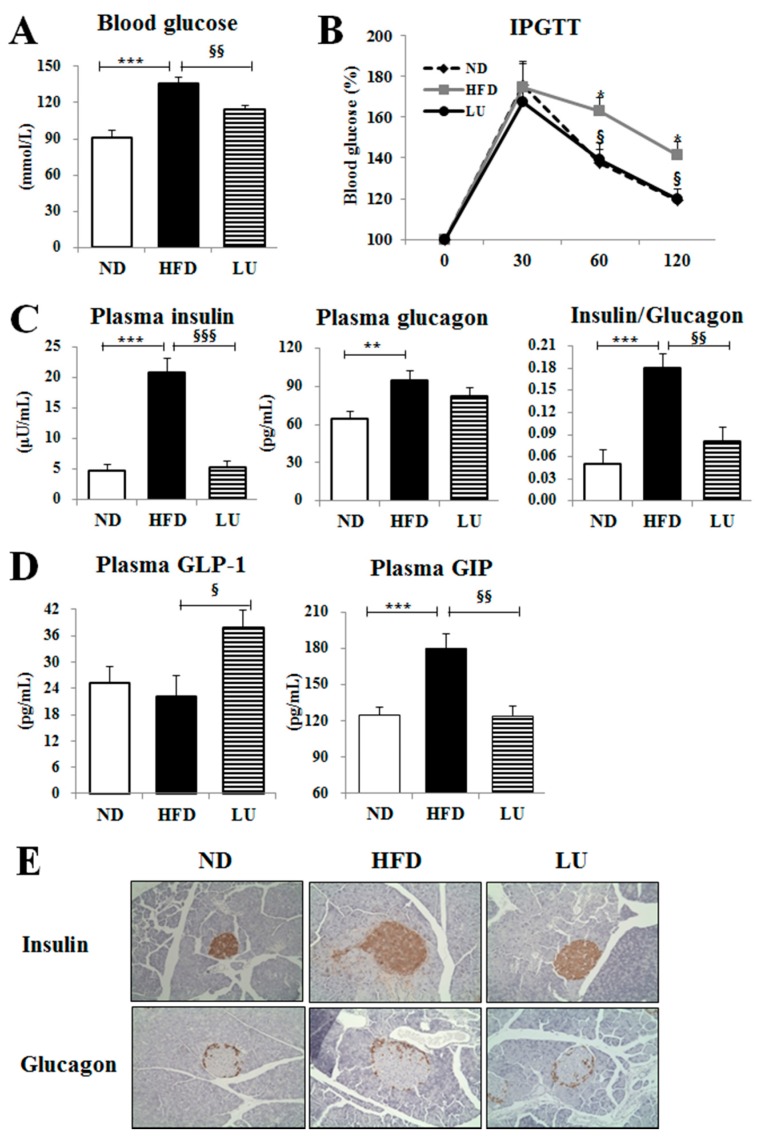
Effect of luteolin treatment for 16 weeks on insulin resistance (IR)-related markers, and immunohistochemistry staining of pancreatic insulin and glucagon in C57BL/6J mice fed the high-fat diet. (**A**) Blood glucose levels after 12 h of fasting. (**B**) IPGTT after 12 h of fasting. (**C**) Plasma levels of insulin and glucagon, and ratio of insulin and glucagon. (**D**) The plasma levels of incretin hormones GLP-1 and GIP. (**E**) Immunohistochemistry staining for insulin and glucagon of the pancreas (200× magnification). Data are shown as means ± SEM. ND vs HFD: * *p* < 0.05, ** *p* < 0.01, *** *p* < 0.001. HFD vs LU: ^§^ *p* < 0.05, ^§§^ *p* < 0.01, ^§§§^ *p* < 0.001.

Luteolin treatment significantly decreased the fasting blood glucose level and improved glucose intolerance, as evidenced by the results of the IPGTT, compared with those of the HFD group (Figure 3A,B). Furthermore, the levels of plasma insulin and glucagon and the ratio of insulin to glucagon were significantly reduced by luteolin treatment compared with those in the HFD group (Figure 3C). Luteolin markedly increased the plasma GLP-1 level but decreased the plasma GIP level (Figure 3D). Immunohistochemistry staining of the pancreatic tissue for insulin and glucagon also revealed that luteolin treatment improved the pancreatic islet hypertrophy caused by the HFD, resulting in a decrease in pancreatic insulin and glucagon content, consistent with the results for plasma insulin and glucagon levels (Figure 3E).

### 3.4. Luteolin Altered the Transcriptional Responses in the Epididymal WAT of DIO Mice

**Figure 4:**
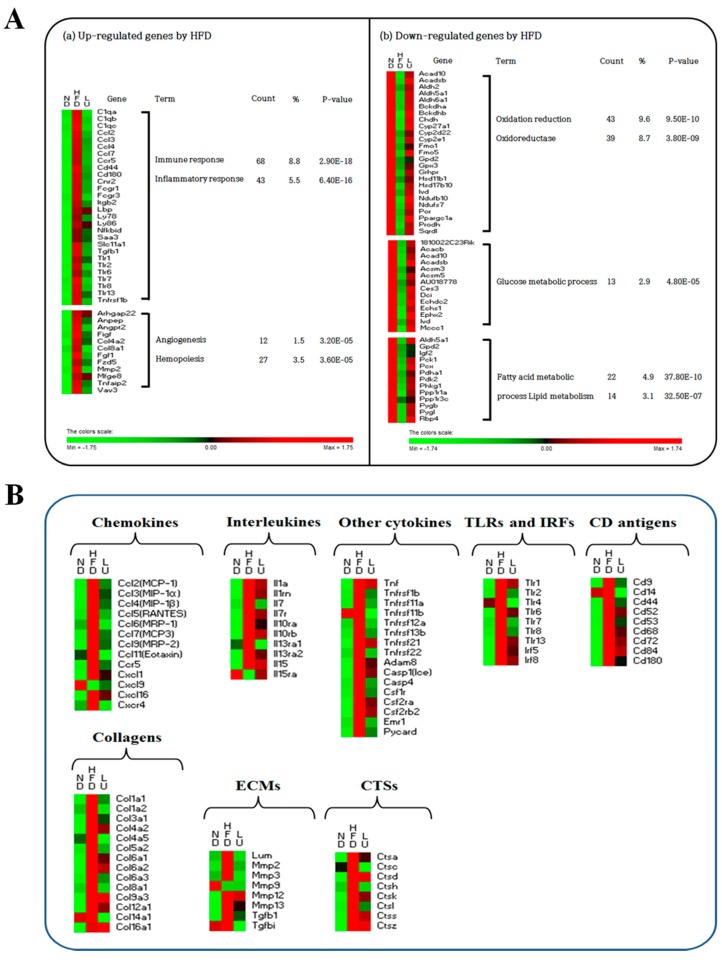
Gene transcription patterns related to inflammation in white adipose tissue. Symbols in red were up-regulated while those in green were down-regulated. (**A**) Functional gene ontology terms enriched among up- or down-regulated genes by the HFD using DAVID (enrichment score >2). (**B**) The heatmap of genes involved in chemokines, interleukins, cytokines, TLRs, IRFs, CD antigens, collagens, ECMs, and CTSs.

The adipocyte microarray analysis showed that 523 genes were differentially expressed in response to luteolin treatment compared with the HFD, including 188 up-regulated genes and 335 down-regulated genes (Table S2). Functional gene ontology terms associated with these luteolin-responsive genes in the epididymal WAT depot were clustered using DAVID (Figure 4A). Luteolin decreased the expression levels of genes associated with immune and inflammatory system function and angiogenesis and hemopoiesis system function that were up-regulated by the HFD. However, functional gene ontology terms related to oxidation reduction as well as glucose and lipid metabolism were enriched among the genes that were up-regulated by luteolin treatment.

As shown in Figure 4B and Tables S3–S8, during the development of diet-induced obesity, adipocyte transcription of several pro-inflammatory chemokines (*Ccl2-7*, *Ccl9*, *Ccl11*, *Ccr5*, *Cxcl1*, *Cxcl16*, and *Cxcr4*), interleukins (*Il1a*, *Il1rn*, *Il7*, *Il7r*, *Il10ra*, *Il10rb*, *Il13ra1*, *Il13ra2*, and *Il15*), and cytokines (*Tnf*, *Tnfrsf1b*, *Tnfrsf11a* and *11b*, *Tnfrsf12a*, *Tnfrsf13b*, *Tnfrsf21*, *Tnfrsf22*, *Adam8*, *Casp1*, *Casp4*, *Csf1r*, *Csf2ra*, *Csf2rb2*, *Saa3*, *Emr1*, and *Pycard*) was significantly decreased, while the expression levels of anti-inflammatory genes such as *Cxcl9* and *Il15ra* were elevated in comparison to levels detected in the HFD group (Figure 4B and Table S3). Moreover, the expression of TLR genes and interferon regulatory factors 5 and 8 (*Irf5* and *Irf8*) was also significantly altered by luteolin treatment. Specifically, *Tlr1*, *Tlr2*, *Tlr4*, *Tlr6*, *Tlr7*, *Tlr8*, *Tlr13*, *Irf5*, and *Irf8* were significantly down-regulated, while *Tlr5* was markedly up-regulated by luteolin (Figure 4B and Table S4). The expression of most of the genes in the CD antigen family was up-regulated by the HFD, including *Cd6*, *Cd9*, *Cd14*, *Cd22*, *Cd33*, *Cd37*, *Cd40*, *Cd44*, *Cd52*, *Cd53*, *Cd68*, *Cd72*, *Cd74*, *Cd83*, *Cd84*, *Cd86*, *Cd93*, *Cd180*, *Cd248*, and *Cd276*, whereas these genes were down-regulated by luteolin treatment (Figure 4B and Table S5). Expression levels of the M2 macrophage markers *Cd163*, *Cd209a*, and *Cd209b* were decreased by the HFD and further down-regulated by luteolin. Interestingly, *Cd36* gene expression, which is responsible for lipid uptake, was increased by luteolin treatment.

Next, we examined the levels of many key fibrotic genes such as adipose tissue-expressed collagen, ECM, and cathepsin family genes during HFD-induced inflammation. The mRNA levels of collagen-related genes that were up-regulated by the HFD, including *Col1a1*, *Col1a2*, *Col3a1*, *Col4a2*, *Col4a5*, *Col5a2*, *Col6a1*, *Col6a2*, *Col6a3*, *Col8a1*, *Col9a3*, *Col12a1*, *Col14a1*, and *Col16a1*, were down-regulated by luteolin (Figure 4B and Table S6). In particular, luteolin treatment lowered the expression of ECM genes, including *Lum*, *Mmp2*, *Mmp3*, *Mmp12*, *Mmp13*, *Tgfb1*, and *Tgfbi* (Figure 4B and Table S7), and cathepsin genes (*Ctsa*, *Ctsc*, *Ctsd*, *Ctsk*, *Ctsl*, and *Ctss*; Figure 4B and Table S8) that were up-regulated by the HFD.

**Figure 5:**
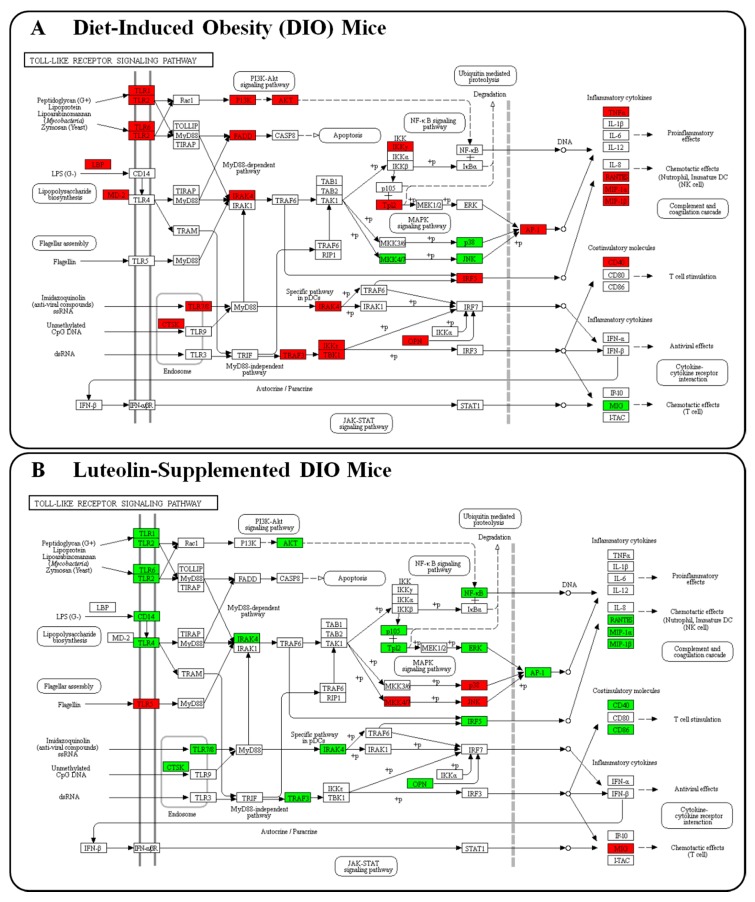
Gene transcription pattern related to the TLR signaling pathway in white adipose tissue. Symbols in red were up-regulated while those in green were down-regulated. (**A**) Gene expression levels altered by the HFD. (**B**) Gene expression levels altered by luteolin treatment.

Moreover, pathway analysis using the KEGG mapper revealed that the TLR signaling pathway was the major pathway altered in response to luteolin treatment during the development of obesity and its complications (Figure 5). Overall, the expression of genes involved in the TLR signaling pathway was up-regulated by the HFD and down-regulated by luteolin. Interestingly, the expression of *Tlr5*, *Mkk4/7*, *p38*, *Jnk*, and *Mig* in the TLR signaling pathway was up-regulated by luteolin but down-regulated by the HFD. In our previous study, we analyzed time-course microarray data of the adipose tissue in DIO mice to elucidate the mechanisms underlying DIO development [18] and found that *Tlr5*, *Map2k7* (Mkk4/7), *Mapk12* and *13*(p38), *Mapk9*, *Mapk8ip1* (Jnk), and *Cxcl9* (Mig) expression was consistently down-regulated by the HFD at most time points except at the early stage (between weeks 2 and 4 of DIO development) (Figure S1). These results are consistent with the changes detected in TLR-related genes in the adipose tissue of HFD-fed mice in the present study.

### 3.5. Luteolin Normalized Epididymal WAT Depot Genes Altered by Advanced Age and Diet-Induced Obesity

**Figure 6:**
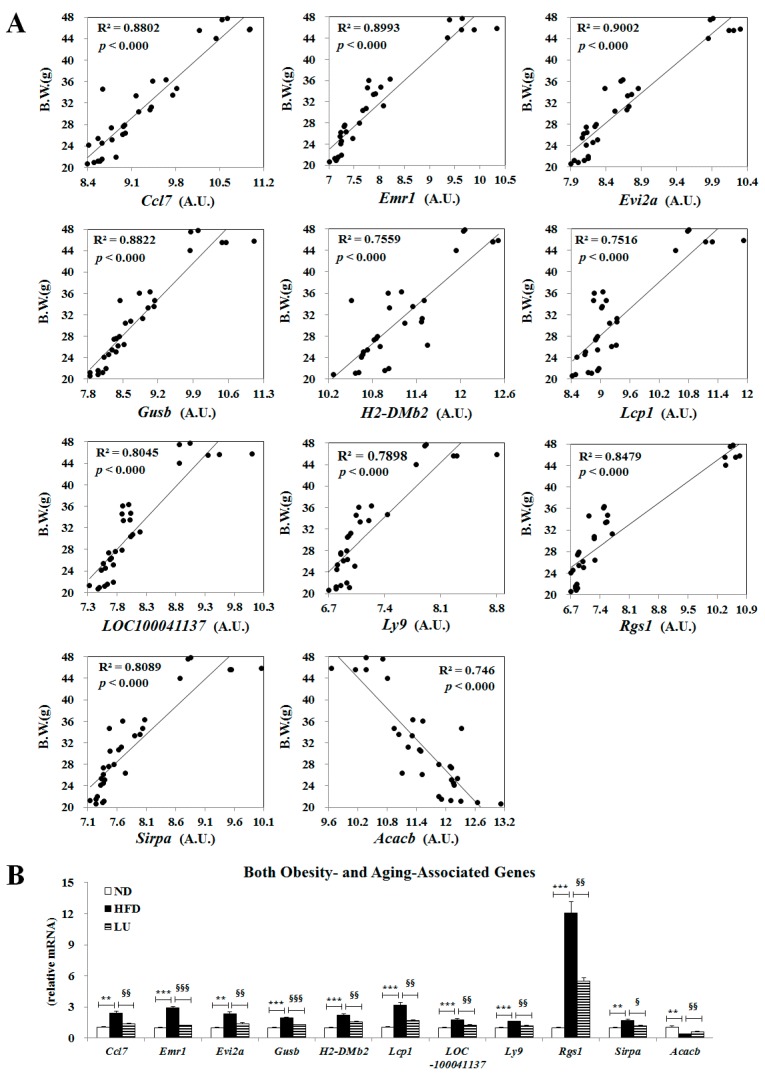
Effect of luteolin treatment for 16 weeks on the expression of genes involved in both obesity and aging. (**A**) Correlation between body weight and genes affected by advancing age (*Ccl7*, *Emr1*, *Evi2a*, *Gusb*, *H2-DMb2*, *Lcp1*, *LOC10041137*, *Ly9*, *Rgs1*, *Sirpa* and *Acacb*). (**B**) Expression of obesity- and aging-associated genes. Data are shown as means ± SEM. ND vs HFD: * *p* < 0.05, ** *p* < 0.01, *** *p* < 0.001. HFD vs LU: ^§^ *p* < 0.05, ^§§^ *p* < 0.01, ^§§§^ *p* < 0.001.

In our previous study, we reported that 14 adipocyte genes (*Ccl7*, *Emr1*, *Evi2a*, *Gusb*, *H2-DMb2*, *Lcp1*, *LOC100041137*, *Ly9*, *Ptpre*, *Rgs1*, *sc1000528.1_16*, *Sirpa*, *Sfrp5*, and *Acacb*) were associated with both advanced age and diet-induced obesity. In line with our previous study, among these genes, *Ccl7*, *Emr1*, *Evi2a*, *Gusb*, *H2-DMb2*, *Lcp1*, *LOC100041137*, *Ly9*, *Rgs1*, and *Sirpa* expression levels were positively correlated with body weight, while the Acacb expression level was negatively correlated with body weight in the present study (Figure 6A). Accordingly, luteolin normalized the expression of these 11 genes altered by the HFD and aging (Figure 6B).

## 4. Discussions and Conclusions

In the current study, we evaluated the protective role of luteolin on inflammation-mediated metabolic diseases, focusing on its role in modulating the TLR-signaling-pathway-triggered up-regulation of pro-inflammatory cytokines.

Excessive adipose tissue is linked with increased angiogenesis, macrophage infiltration, production of ECM components, and the production and release of several inflammatory mediators, which cause a state of chronic low-grade inflammation and promote obesity-linked metabolic disorders such as IR [19,20]. In this study, HFD led to the dysregulation of pro- and anti-inflammatory cytokines/adipokines, macrophage infiltration, and ECM accumulation. Luteolin treatment significantly lowered the plasma levels of pro-inflammatory adipokines (leptin and resistin) and cytokines (TNF-α, MCP-1, IL-6, MIP-1β, PAI-1, and adipsin), and increased the anti-inflammatory cytokine adiponectin level, thereby improving adipokine dysregulation coupled with adiposity. In particular, luteolin dramatically lowered the plasma sCD163 level, which is an important marker of macrophage activation [21,22,23,24], with a simultaneous decrease in the expression levels of monocyte/macrophage markers (*Cd68*, *Ccl2*, *Emr1*, *Csf1r*, and *Saa3*) in adipocytes; this, in turn, induced a significant decrease in hepatic and adipocyte macrophage infiltration and accumulation as evidenced by immunohistochemical staining of CD68 and MCP-1.

Oligonucleotide microarray profiling also revealed that luteolin treatment down-regulated the expression of adipocyte genes involved in inflammation compared with the effects of the HFD. Luteolin markedly decreased the mRNA expression levels of pro-inflammatory chemokines, interleukins, and cytokines along with their upstream signaling pathway genes such as TLRs, CDs, and IRFs, including *Tlr4*, *Cd14*, and *Irf5*, all of which are used as markers of visceral adipose tissue inflammation and linked with obesity and/or IR [18,25]. Furthermore, among the pro-inflammatory genes that were down-regulated by luteolin treatment, *Emr1* and *Ccl7* are associated with advanced age as well as obesity-related inflammation. Adipocyte *Emr1* and *Ccl7* mRNA expression levels increased with advancing age in the normal diet group and were further up-regulated when given the HFD; however, luteolin decreased the *Emr1* and *Ccl7* mRNA expression levels in proportion to body weight compared with the levels in the HFD group. Thus, the *Emr1* and *Ccl7* genes could be targets for ameliorating the deleterious effects of the obesity-induced inflammation response since these genes are not only very sensitive to body weight but also important factors inducing chronic low-grade inflammation. It is plausible that luteolin prevented adipose tissue expansion, the concomitant activation of TLR-mediated inflammatory signaling cascades, and induction of CD antigens. Luteolin consequently decreased the expression levels of pro-inflammatory cytokines, chemokines, and interleukins, thereby ameliorating the chronic low-grade inflammation in DIO mice. Interestingly, luteolin treatment led to an increase in the expression of *Tlr5* (TLR5), *Map2k7* (MKK7), *Mapk12* and *Mapk13* (p38), and *Mapk9* (JNK) genes, although it decreased the overall expression of genes involved in the TLR signaling pathway, suggested as a major pathway in response to luteolin treatment; these gene expression patterns were reversed by the HFD. Previous studies have indicated a positive role for p38, MKK, and JNK in inducing adipogenesis [26,27,28]. Our previous study suggested that an HFD decreased FA synthesis as well as FA oxidation due to the adaptation to overnutrition, and luteolin activated adipocyte lipogenesis to protect other organs from ectopic fat accumulation and, consequently, from lipotoxicity [29]. Moreover, Vijay-Kumar et al. [30] reported that Tlr5^−/−^ mice exhibit hyperphagia and develop hallmark features of metabolic syndrome, including dyslipidemia and IR. Therefore, our present observation suggests that p38, MKK, and JNK could be associated with lipogenesis rather than inflammation in the TLR signaling pathway, and that *Tlr5* may be a key transcription factor for luteolin-mediated lipotoxicity protection.

The anti-inflammatory effects coupled with the decrease in adiposity conferred by luteolin were strongly linked to the improvement of fibrosis in the adipose tissue as evidenced by the results of Masson’s trichrome staining. Fibrosis, attributed to the excessive deposition of ECM proteins, including collagen, is a ubiquitous tissue response to chronic inflammation [18,31]. Furthermore, cathepsin family members are involved in the remodeling of ECM proteins, and have been implicated in various ECM-related disorders such as fibrosis [3,4,5,6,7,32]. In this study, luteolin treatment down-regulated collagen mRNA levels, which may trigger the reduction in the expression of cathepsin family proteins and ECM accumulation to ultimately lead to the attenuation of fibrosis, which was evident in diet-induced obesity. Luteolin also increased the activities of hepatic-hydrogen-peroxide-detoxifying enzymes (catalase and GPx), resulting in reduced hepatic mitochondrial H_2_O_2_ and TBARS levels, which reflects improved hepatic lipotoxicity along with reduced plasma GOT and GPT levels. The inflammatory response is complex and often involves reactive oxygen species (ROS) such as H_2_O_2_ [33]. ROS-induced oxidative stress results in dysregulated pro-inflammatory cytokine production, which triggers the fibrogenesis signaling pathways and subsequently promotes the progression of steatosis to nonalcoholic steatohepatitis [34]. Thus, a luteolin-induced decrease in macrophage infiltration and ECM accumulation in the adipose tissue and a decrease in hepatic oxidative stress would be expected to improve chronic low-grade inflammation via the interplay between liver and adipose tissue, eventually improving fibrosis.

The improved chronic low-grade inflammation by luteolin through modulation of the TLR signaling pathway was likely associated with a normalization of pancreatic beta-cell mass and insulin levels, leading to the prevention of IR. IR is always associated with inflammation with regard to adiposopathy. Adipokine/cytokine dysregulation caused by adiposopathy impairs insulin secretion and action in pancreatic beta-cells and induces global IR [35]. Moreover, in conditions of adiposopathy, ROS produce significant amounts of inflammatory cytokines and angiogenic factors to inhibit the production of adiponectin by white adipocytes; thus, the adipose tissue cannot store the surplus of FFAs, further contributing to the development of IR [36]. Expansion of pancreatic beta-cell mass can also result in IR by increasing proliferation and hypertrophy, as well as the amount of insulin secretion per beta-cell to compensate for insulin desensitizing [37]. FFAs are an important link between adiposopathy and IR by acting as insulin secretagogues in islet beta-cells and inducing lipotoxicity. As mentioned above, luteolin protected against hepatic and pancreatic lipotoxicity via increasing FA uptake, lipogenesis, and FA oxidation in the adipose tissue with high capacity for the detoxification of FA. In addition, increases in p38-, MKK-, and JNK-mediated lipogenesis by luteolin on the TLR signaling pathway can be partially affected by the increased *Tlr5* expression. Moreover, luteolin significantly increased the plasma GLP-1 level but decreased the plasma GIP level compared with the HFD, leading to amelioration of IR. The two incretin hormones GLP-1 and GIP have dissimilar metabolic effects, with GLP-1 being more favorable for glucose and lipid homeostasis, as GLP-1 inhibits glucagon, increases insulin sensitivity and action, and has an anti-inflammatory effect in the liver against steatosis [38]. In contrast, GIP is involved in energy storage and fat deposition, which promotes obesity-induced IR development [38,39].

**Figure 7:**
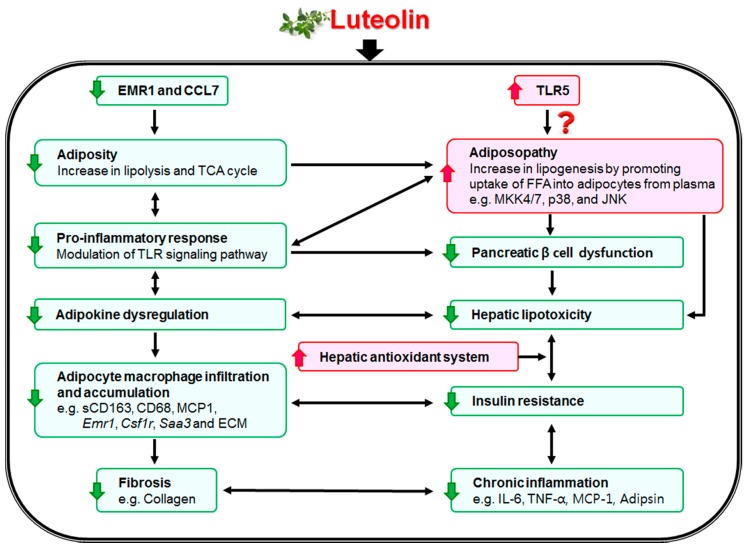
Proposed role of luteolin in attenuating hepatic and adipocyte fibrosis and insulin resistance by targeting the Toll-like receptor signaling pathway in high-fat-fed mice. Luteolin improved chronic low-grade inflammation by modulating the TLR signaling pathway, resulting in suppression of hepatic and adipocyte fibrosis by decreasing pro-inflammatory cytokines, adipokine dysregulation, macrophage and extracellular matrix accumulation, and captesin gene expressions. Interestingly, *Emr1* and *Ccl7*, important markers inducing low-grade inflammation, were affected by advanced age and greater body weight, which were normalized by luteolin treatment. Moreover, luteolin prevented hepatic lipotoxicity via an increase in uptake of FFA into adipocytes from plasma [29] and the hepatic antioxidant system, which was linked to activation of lipogenesis in adipose tissue mediated via *Tlr5*, *Map2k7*, *Mapk12*, *Mapk13*, and *Mapk9* gene expressions; further, *Tlr5* may be a key transcription factor for luteolin-mediated lipotoxicity protection. Decreased plasma FFA levels by luteolin were associated with improved pancreatic islet dysfunction, which all contribute to improvement of lipotoxicity, fibrosis, and insulin resistance.

In summary, the data obtained from our animal study indicate that luteolin can suppress diet-induced obesity and modulate obesity-associated metabolic disorders. Luteolin decreased adipokine/cytokine dysregulation, macrophage infiltration, and ECM accumulation in the adipose tissue through modulating the TLR signaling pathway, and reduced excessive ROS production via an increase in hepatic antioxidant capacity. Luteolin improved and normalized pancreatic islet dysfunction by differentially regulating plasma GLP-1 and GIP, which contributes to improvement of lipotoxicity, fibrosis, and IR. The noticeable improvement in lipotoxicity by luteolin is linked with increased adipocyte lipogenesis mediated via p38, MKK, and JNK; *Tlr5* is a possible key transcription factor for preventing lipotoxicity, although this observation requires additional experiments for verification. Figure 7 illustrates the possible mechanisms of the effects of luteolin on hepatic and adipocyte fibrosis and IR. Taken together, the present findings suggest that luteolin ameliorates the deleterious effects of diet-induced obesity and its metabolic complications such as adiposopathy, fibrosis, and IR.

## Figures and Tables

**Table 1:** Effect of luteolin treatment for 16 weeks on body and organ weights, and plasma and hepatic FFA levels in C57BL/6J mice fed the high-fat diet.

	ND	HFD	LU
Initial Body Weight (g)	19.33 ± 0.25	19.33 ± 0.32	19.33 ± 0.29
Final Body Weight (g)	30.32 ± 0.78	42.17 ± 0.89 ***	37.48 ± 0.79 ^§§§^
Body Weight Gain (g/weeks)	0.70 ± 0.07	1.47 ± 0.07 ***	1.12 ± 0.07 ^§§§^
Liver (g/100 g BW)	3.54 ± 0.13	6.57 ± 0.20 ***	5.87 ± 0.30
Visceral WAT (g/100 g BW)	5.83 ± 0.31	9.63 ± 0.19 ***	7.33 ± 0.24 ^§§§^
Subcutaneous WAT (g/100 g BW)	1.62 ± 0.10	3.65 ± 0.10 ***	2.51 ± 0.05 ^§§§^
Total WAT (g/100 g BW)	8.62 ± 0.52	15.31 ± 0.27 ***	11.68 ± 0.20 ^§§§^
Plasma FFA (mmol/L)	1.13 ± 0.07	1.43 ± 0.04 **	1.15 ± 0.02 ^§§§^
Hepatic FFA (mEq/g liver)	0.014 ± 0.001	0.028 ± 0.001 ***	0.020 ± 0.001 ^§§§^

Data are shown as means ± SEM. ND vs HFD: * *p* < 0.05, ** *p* < 0.01, *** *p* < 0.001. HFD vs LU: ^§^ *p* < 0.05, ^§§^ *p* < 0.01, ^§§§^ *p* < 0.001. BW, body weight; WAT, white adipose tissue; FFA, free fatty acid, ND, normal diet, HFD, high-fat diet, LU, Luteolin.

## Supplementary Materials

The following are available online at http://www.mdpi.com/2072-6643/10/10/1415/s1, Figure S1: Effect of high-fat feeding on transcription of TLR5, MKK4/7, p38, JNK and MIG-realted genes in epididymal adipose tissue of C57BL/6 J mice over 24 weeks (HFD vs. ND group). Data shown as means ± S.D. * *p* < 0.05 based on wilcoxon t-test. ND: normal diet. HFD: high-fat diet, Table S1: Primer sequences used for RT-qPCR validation of the microarray data, Table S2: The number of differentially expressed genes (DEGs) in the epididymal WAT of C57BL/6J mice, Table S3: Effect of luteolin on transcriptional pattern of anti- and pro-inflammatory cytokine and chemokine genes in adipose tissue of C57BL/6J mice, Table S4: Effect of luteolin on transcriptional pattern of toll-like receptors (TLRs), interferon regulatory factors (IRFs) and Cd antigen 14 (Cd14) in adipose tissue and liver of C57BL/6J mice, Table S5: Effect of luteolin on transcriptional pattern of Cd antigen families in adipose tissue and liver of C57BL/6J mice, Table S6: Effect of luteolin on transcriptional pattern of collagen in adipose tissue and liver of C57BL/6J mice, Table S7: Effect of luteolin on transcriptional pattern of extracellular matrix (ECM)'s regulator in adipose tissue and liver of C57BL/6J mice, Table S8: Effect of luteolin on transcriptional pattern of cathepsin in adipose tissue and liver of C57BL/6J mice.

## Abbreviations

CAT	catalase
DIO	diet-induced obesity
ECM	extracellular matrix
FFA	free fatty acid
GIP	gastric inhibitory polypeptide
GLP-1	glucagon-like peptide-1
GOT	glutamic oxaloacetic transaminase
GPT	glutamic pyruvic transaminase
GPx	glutathione peroxidase
GR	glutathione reductase
HFD	high-fat diet
IR	insulin resistance
MCP-1	monocyte chemoattractant protein-1
MIP-1β	macrophage inflammatory protein 1 beta
PAI-1	plasminogen activator inhibitor-1
sCD163	soluble CD antigen 163
SOD	superoxide dismutase
TBARS	thiobarbituric acid reactive substances
TLR	Toll-like receptor
WAT	white adipose tissue
