# Supplementary material for: Luteolin Targets the Toll-Like Receptor Signaling Pathway in Prevention of Hepatic and Adipocyte Fibrosis and Insulin Resistance in Diet-Induced Obese Mice
Source: Nutrients. 2018 Oct 3;10(10):1415. doi: 10.3390/nu10101415 (PMC6213163; doi:10.3390/nu10101415)
Supplement: Supplementary file 1 [file nutrients-10-01415-s001.pdf]

Table S1. Primer sequences used for RT-qPCR validation of the microarray data.

Gene	Primer direction	Primer sequence
Glyceraldehyde 3-phosphate dehydrogenase (Gapdh)	Forward	5′-AGGTCGGTGTGAACGGATTTG-3′
	Reverse	5′-TGTAGACCATGTAGTTGAGGTCA-3′
CD antigen 68 (Cd68)	Forward	5′-CCATCCTTCACGATGACACCT-3′
	Reverse	5′-GGCAGGGTTATGAGTGACAGTT-3′
Chemokine (C-C motif) ligand (Ccl2, MCP-1)	Forward	5′-ACTGAAGCCAGCTCTCTCTTCCTC-3′
	Reverse	5′-TTCCTTCTTGGGGTCAGCACAGAC-3′
Egf-like module containing, mucin-like, hormone receptor-like 1 (Emr1, F4/80)	Forward	5′-CCCCAGTGTCCTTACAGAGTG-3′
	Reverse	5′-GTGCCAGAGTGGATGTCT-3′
Colony stimulating factor 1 receptor (Csf1r)	Forward	5′-CCACCATCCACTTGTATGTCAAAGAT-3′
	Reverse	5′-CTCAACCACTGTCACCTCCTGT-3′
Serum amyloid A 3 (Saa3)	Forward	5′-GCCTGGGCTGCTAAAGTCAT-3′
	Reverse	5′-TGCTCCATGTCCCGTGAAC-3′

Table S2. The number of differentially expressed genes (DEGs) in the epididymal WAT of C57BL/6J mice.

	HFD vs. ND	LU vs. HFD
▲	1038	188
▼	640	335

Differentially expressed genes based on comparison of HFD vs. ND and LU vs. HFD according to *p*-value < 0.05, fold change > 1.5 in the epididymal WAT depot.

▲: up-regulated genes, ▼: down-regulated genes.

Table S3. Effect of luteolin on transcriptional pattern of anti- and pro-inflammatory cytokine and chemokine genes in adipose tissue of C57BL/6J mice.

	ND	HFD	LU
Chemokines			
Ccl2 (MCP-1)	1.00 ± 0.04	2.12 ± 0.07***	1.29 ± 0.01§§§
Ccl3 ((MIP-1α)	1.00 ± 0.11	1.68 ± 0.07***	1.26 ± 0.02§§§
Ccl4 (MIP-1β)	1.00 ± 0.01	2.09 ± 0.15***	1.33 ± 0.05§§
Ccl5 (RANTES)	1.00 ± 0.06	1.41 ± 0.21*	1.07 ± 0.05§
Ccl6 (MRP-1)	1.01 ± 0.19	1.57 ± 0.04**	0.91 ± 0.03§§§
Ccl7 (MCP3)	1.01 ± 0.13	2.39 ± 0.33**	1.38 ± 0.04§§
Ccl9 (MRP-2)	1.00 ± 0.09	2.41 ± 0.18***	1.42 ± 0.04§§§
Ccl11 (Eotaxin)	1.00 ± 0.02	1.16 ± 0.11	0.89 ± 0.00§
Ccr5	1.00 ± 0.04	3.04 ± 0.43***	1.52 ± 0.05§§
Cxcl1	1.00 ± 0.08	1.70 ± 0.10***	1.32 ± 0.02§§
Cxcl9	1.00 ± 0.06	0.60 ± 0.02**	0.73 ± 0.02§
Cxcl16	1.00 ± 0.03	1.67 ± 0.10**	1.34 ± 0.02§
Cxcr4	1.01 ± 0.15	1.48 ± 0.08**	0.91 ± 0.02§
Interleukines			
Il1a	1.00 ± 0.03	1.19 ± 0.04*	1.13 ± 0.06
Il1rn	1.00 ± 0.04	3.68 ± 0.37**	2.28 ± 0.06§
Il7	1.00 ± 0.06	1.27 ± 0.02*	1.03 ± 0.01§§§
Il7r	1.00 ± 0.06	6.51 ± 0.60***	3.34 ± 0.23§§

Il10ra	1.00 ± 0.04	1.56 ± 0.06**	1.26 ± 0.02§§
Il10rb	1.00 ± 0.01	1.52 ± 0.04***	1.37 ± 0.09
Il13ra1	1.00 ± 0.04	1.24 ± 0.01**	0.86 ± 0.02§§§
Il13ra2	1.00 ± 0.04	1.85 ± 0.13**	1.43 ± 0.03§
Il15	1.00 ± 0.02	1.31 ± 0.04**	1.24 ± 0.02
Il15ra	1.00 ± 0.06	0.64 ± 0.01**	0.87 ± 0.03§§§
Other cytokines			
Tnf	1.00 ±0.02	1.30 ± 0.05**	1.24 ± 0.05
Tnfrsf1b	1.00 ± 0.03	1.82 ± 0.09***	1.23 ± 0.01§§
Tnfrsf11a	1.00 ± 0.02	1.33 ± 0.04**	1.07 ± 0.06§
Tnfrsf11b	1.01 ± 0.10	1.02 ± 0.05	0.54 ± 0.02§§§
Tnfrsf12a	1.00 ± 0.04	1.31 ± 0.03**	1.02 ± 0.04§§
Tnfrsf13b	1.00 ± 0.04	1.45 ± 0.05***	1.11 ± 0.04§§
Tnfrsf21	1.00 ± 0.06	1.78 ± 0.04***	1.53 ± 0.03§
Tnfrsf22	1.00 ± 0.04	1.30 ± 0.03**	1.03 ± 0.02§§
Adam8	1.00 ± 0.02	2.80 ± 0.20***	1.76 ± 0.03§§
Casp1 (Ice)	1.00 ± 0.03	2.40 ± 0.04***	1.71 ± 0.03§§§
Casp4	1.00 ± 0.03	1.35 ± 0.04**	1.09 ± 0.03§§
Csf1r	1.00 ± 0.05	1.83 ± 0.09***	1.23 ± 0.09§§
Csf2ra	1.00 ± 0.03	2.09 ± 0.13***	1.55 ± 0.09§
Csf2rb2	1.00 ± 0.01	2.00 ± 0.13**	1.58 ± 0.03§
Saa3	1.00 ± 0.02	9.44 ± 1.09***	5.78 ± 0.61§
Emr1	1.00 ± 0.02	2.93 ± 0.12***	1.21 ± 0.00§§§
Pycard	1.00 ± 0.04	1.39 ± 0.01***	1.12 ± 0.04§§

Data shown as means ± S.E. ND vs HFD: $^{*}p < 0.05$, $^{**}p < 0.01$, $^{***}p < 0.001$. HFD vs LU: $^{§}p < 0.05$, $^{§§}p < 0.01$, $^{§§§}p < 0.001$. Ccl, chemokine (C-C motif) ligand; Ccr, chemokine (C-C motif) receptor; Cxcl, chemokine (C-X-C motif) ligand; Cxcr, chemokine (C-X-C motif) receptor, Il1rn, interleukin 1 receptor antagonist; Il, interleukin; Ilr, interleukin receptor; Tnf, tumor necrosis factor; Tnfrsf, tumor necrosis factor receptor; Adam8, a disintegrin and metallopeptidase domain 8; Casp, caspase; Csfr, colony stimulating factor receptor; Saa, serum amyloid A; Emr1, egf-like module containing, mucin-like, hormone receptor-like 1; Pycard, PYD and CARD domain containing.

Table S4. Effect of luteolin on transcriptional pattern of toll-like receptors (TLRs), interferon regulatory factors (IRFs) and Cd antigen 14 (Cd14) in adipose tissue and liver of C57BL/6J mice.

	**ND**	**HFD**	**LU**
Tlr1	1.00 ± 0.04	2.23 ± 0.06***	1.74 ± 0.01§§
Tlr2	1.00 ± 0.04	1.66 ± 0.04***	1.17 ± 0.02§§§
Tlr4	1.00 ± 0.04	1.15 ± 0.04	0.82 ± 0.02§§
Tlr5	1.01 0.05	1.04 0.03	1.25 0.02§§
Tlr6	1.00 ± 0.03	1.61 ± 0.03***	1.31 ± 0.02§§§
Tlr7	1.00 ± 0.06	1.78 ± 0.04***	1.10 ± 0.00§§§
Tlr8	1.01 ± 0.07	2.21 ± 0.09***	1.29 ± 0.02§§§
Tlr13	1.00 ± 0.06	5.05 ± 0.46***	2.66 ± 0.16§§
Irf5	1.00 ± 0.04	2.21 ± 0.09***	1.55 ± 0.05§§
Irf8	1.00 ± 0.06	1.91 ± 0.13**	1.51 ± 0.05§
Cd14	1.01 ± 0.08	1.10 ± 0.02	0.69 ± 0.01§§§

Data shown as means ± S.E. ND vs HFD: $^{*}p < 0.05$, $^{**}p < 0.01$, $^{***}p < 0.001$. HFD vs LU: $^{§}p < 0.05$, $^{§§}p < 0.01$, $^{§§§}p < 0.001$.

Table S5. Effect of luteolin on transcriptional pattern of Cd antigen families in adipose tissue and liver of C57BL/6J mice.

	ND	HFD	LU
Cd6	1.00 ± 0.01	1.34 ± 0.08*	1.02 ± 0.01§
Cd9	1.00 ± 0.02	2.35 ± 0.17**	1.39 ± 0.05§§
Cd14	1.01 ± 0.09	1.10 ± 0.02	0.69 ± 0.01§§§
Cd22	1.00 ± 0.05	1.45 ± 0.04**	1.26 ± 0.03§
Cd33	1.00 ± 0.05	1.01 ± 0.02	0.78 ± 0.02§§§
Cd36	1.00 ± 0.04	1.05 ± 0.05	1.59 ± 0.04§§§
Cd37	1.00 ± 0.04	1.53 ± 0.09**	1.16 ± 0.02§
Cd40	1.01 ± 0.08	1.47 ± 0.07*	1.25 ± 0.02§
Cd44	1.00 ± 0.01	3.42 ± 0.24***	1.67 ± 0.06§§
Cd52	1.00 ± 0.06	2.38 ± 0.14***	1.42 ± 0.01§§
Cd53	1.00 ± 0.07	2.42 ± 0.19**	1.51 ± 0.16§
Cd68	1.01 ± 0.12	4.90 ± 0.43***	2.42 ± 0.05§§
Cd72	1.00 ± 0.03	4.15 ± 0.40***	2.43 ± 0.11§
Cd74	1.00 ± 0.07	1.68 ± 0.05***	1.10 ± 0.02§§§
Cd83	1.01 ± 0.09	1.48 ± 0.11*	0.88 ± 0.03§§
Cd84	1.01 ± 0.08	4.79 ± 0.34***	2.60 ± 0.04§§
Cd86	1.00 ± 0.03	1.08 ± 0.01	0.83 ± 0.01§§§
Cd93	1.00 ± 0.03	1.41 ± 0.03***	1.12 ± 0.03§§
Cd163	1.00 ± 0.05	0.69 ± 0.05*	0.53 ± 0.01§
Cd180	1.00 ± 0.02	3.10 ± 0.29**	1.74 ± 0.03§§
Cd209a	1.00 ± 0.03	0.87 ± 0.03*	0.66 ± 0.01§§
Cd209b	1.01 ± 0.07	0.65 ± 0.02**	0.37 ± 0.01§§§
Cd248	1.00 ± 0.03	1.20 ± 0.09	0.78 ± 0.01§§
Cd276	1.01 ± 0.07	1.79 ± 0.06***	1.35 ± 0.03§§

Data shown as means ± S.E. ND vs HFD: $^{*}p < 0.05$, $^{**}p < 0.01$, $^{***}p < 0.001$. HFD vs LU: $^{§}p < 0.05$, $^{§§}p < 0.01$, $^{§§§}p < 0.001$.

Table S6. Effect of luteolin on transcriptional pattern of collagen in adipose tissue and liver of C57BL/6J mice.

	ND	HFD	LU
Col1a1	1.01 ± 0.08	2.03 ± 0.14**	1.26 ± 0.02§§
Col1a2	1.00 ± 0.03	1.40 ± 0.05**	0.97 ± 0.04§§
Col3a1	1.01 ± 0.09	2.64 ± 0.10***	1.55 ± 0.05§§§
Col4a1	1.00 ± 0.05	1.34 ± 0.06*	1.07 ± 0.05§
Col4a2	1.00 ± 0.05	1.69 ± 0.08**	1.40 ± 0.03§
Col4a5	1.01 ± 0.11	1.27 ± 0.03	0.85 ± 0.01§§§
Col5a2	1.00 ± 0.05	1.46 ± 0.10*	1.08 ± 0.05§
Col6a1	1.00 ± 0.06	1.68 ± 0.06***	1.34 ± 0.03§§
Col6a2	1.01 ± 0.12	1.95 ± 0.09**	1.62 ± 0.05§
Col6a3	1.00 ± 0.07	1.74 ± 0.11**	1.19 ± 0.02§§
Col8a1	1.00 ± 0.05	1.42 ± 0.07**	1.00 ± 0.01§§
Col9a3	1.00 ± 0.04	1.62 ± 0.05***	1.59 ± 0.08
Col12a1	1.00 ± 0.06	3.27 ± 0.04***	2.13 ± 0.05§§§
Col14a1	1.00 ± 0.05	1.07 ± 0.06	0.65 ± 0.01§§
Col16a1	1.02 ± 0.14	2.32 ± 0.04***	2.47 ± 0.09

Data shown as means ± S.E. ND vs HFD: $^{*}p < 0.05$, $^{**}p < 0.01$, $^{***}p < 0.001$. HFD vs LU: $^{§}p < 0.05$, $^{§§}p < 0.01$, $^{§§§}p < 0.001$.

Table S7. Effect of luteolin on transcriptional pattern of extracellular matrix (ECM)'s regulator in adipose tissue and liver of C57BL/6J mice.

	ND	HFD	LU
Cd44	1.00 ± 0.01	3.42 ± 0.23***	1.67 ± 0.06§§

Lum	1.02 ± 0.14	2.21 ± 0.10**	0.93 ± 0.02§§§
Mmp2	1.02 ± 0.14	1.86 ± 0.10**	1.11 ± 0.02§§
Mmp3	1.00 ± 0.01	1.62 ± 0.05***	0.93 ± 0.06§§§
Mmp9	1.01 ± 0.11	0.61 ± 0.01*	0.62 ± 0.02
Mmp12	1.01 ± 0.08	15.14 ± 1.43***	9.85 ± 0.73§
Mmp13	1.01 ± 0.09	2.03 ± 0.19**	1.44 ± 0.11§
Tgfb1	1.00 ± 0.02	1.49 ± 0.02***	1.19 ± 0.02§§§
Tgfbi	1.00 ± 0.01	1.20 ± 0.04*	0.76 ± 0.00§§§

Data shown as means ± S.E. ND vs HFD: $^{*}p < 0.05$, $^{**}p < 0.01$, $^{***}p < 0.001$. HFD vs LU: $^{§}p < 0.05$, $^{§§}p < 0.01$, $^{§§§}p < 0.001$. Cd44, CD antigen 44; Lum, lumican; Mmp, matrix metalloproteinases; Tgfb, transforming growth factor beta.

Table S8. Effect of luteolin on transcriptional pattern of cathepsin in adipose tissue and liver of C57BL/6J mice.

	ND	HFD	LU
Ctsa	1.01 ± 0.09	2.40 ± 0.12***	1.60 ± 0.02§§
Ctsc	1.00 ± 0.06	1.17 ± 0.03	0.86 ± 0.01§§§
Ctsd	1.00 ± 0.03	1.43 ± 0.01***	1.40 ± 0.04
Ctsh	1.00 ± 0.02	0.63 ± 0.02***	0.92 ± 0.03§§§
Ctsk	1.01 ± 0.07	4.60 ± 0.32***	2.58 ± 0.09§§
Ctsl	1.00 ± 0.02	1.82 ± 0.10***	1.23 ± 0.05§§
Ctss	1.01 ± 0.11	3.74 ± 0.15***	2.63 ± 0.12§§
Ctsz	1.01 ± 0.07	1.58 ± 0.10**	1.61 ± 0.08
Col12a1	1.00 ± 0.06	3.27 ± 0.04***	2.13 ± 0.05§§§
Col14a1	1.00 ± 0.05	1.07 ± 0.06	0.65 ± 0.01§§
Col16a1	1.02 ± 0.14	2.32 ± 0.04***	2.47 ± 0.09

Data shown as means ± S.E. ND vs HFD: $^{*}p < 0.05$, $^{**}p < 0.01$, $^{***}p < 0.001$. HFD vs LU: $^{§}p < 0.05$, $^{§§}p < 0.01$, $^{§§§}p < 0.001$. Cts, Cathpsin.

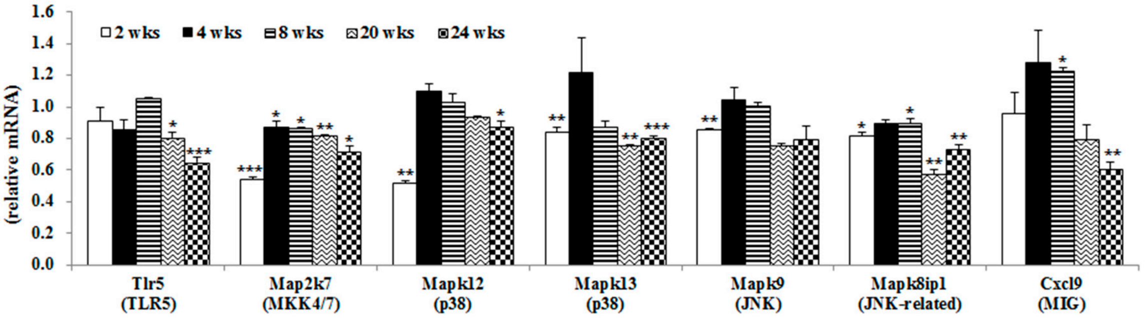

Figure S1. Effect of high-fat feeding on transcription of TLR5, MKK4/7, p38, JNK and MIG-realted genes in epididymal adipose tissue of C57BL/6 J mice over 24 weeks (HFD vs. ND group). Data shown as means ± S.D. * $p < 0.05$ based on wilcoxon t-test. ND: normal diet. HFD: high-fat diet.
